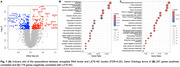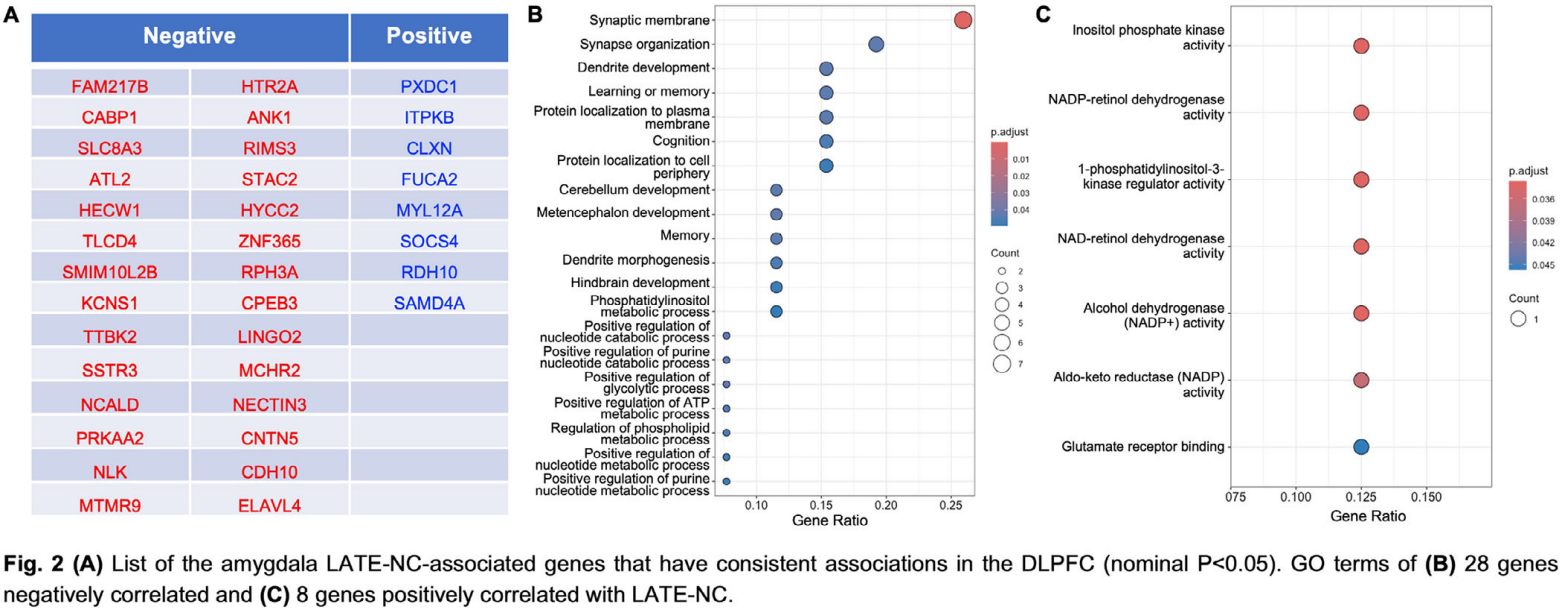# Molecular signatures of LATE‐NC: insights from transcriptomic analyses

**DOI:** 10.1002/alz70856_098456

**Published:** 2025-12-24

**Authors:** Jijing Wang, Ling Teng, Sashini L De Tissera, Nicola Kearns, Zheng Yin, David H. Adamowicz, Jishu Xu, Lei Liu, Jasmeer P. Chhatwal, Philip L. De Jager, Yanling Wang, David A. A. Bennett, Vilas Menon, Vladislav A Petyuk, Julie A Schneider, Hyun‐Sik Yang

**Affiliations:** ^1^ Mass General Brigham, Boston, MA, USA; ^2^ Harvard Medical School, Boston, MA, USA; ^3^ The Broad Institute of MIT and Harvard, Cambridge, MA, USA; ^4^ Rush Alzheimer's Disease Center, Rush University Medical Center, Chicago, IL, USA; ^5^ Columbia University Irving Medical Center, New York, NY, USA; ^6^ Rush Alzheimer's Disease Center, Chicago, IL, USA; ^7^ Pacific Northwest National Laboratory, Richland, WA, USA; ^8^ Department of Neurology, Harvard Medical School, Boston, MA, USA

## Abstract

**Background:**

Limbic‐predominant age‐related TDP‐43 encephalopathy neuropathological change (LATE‐NC) is the third most significant contributor to late‐onset dementia after Alzheimer's disease neuropathologic change (ADNC; amyloid‐β and tau) and cerebrovascular disease. However, the molecular alterations underlying LATE‐NC remain poorly understood.

**Method:**

We studied 959 participants (Age=89.4±6.7, Female: 65.9%) from Religious Orders Study and the Rush Memory and Aging Project (ROSMAP). Bulk RNA sequencing (RNA‐seq) was performed on post‐mortem brain tissues from the amygdala (*n* = 97) and dorsolateral prefrontal cortex (DLPFC; *n* = 937), with 75 overlapping participants. Linear regression models were used to evaluate associations between RNA expression and LATE‐NC burden (TDP‐43 immunohistochemistry; the average semi‐quantitative counts of neuronal cytoplasmic inclusions across six brain regions), adjusting for age, sex, and quantitative ADNC burden (amyloid‐β and tau across eight brain regions). Gene Ontology (GO) terminology overrepresentation was assessed for genes with positive or negative associations with LATE‐NC.

**Results:**

RNA‐seq analysis of the amygdala (*n* = 97) identified 257 genes positively correlated and 178 genes negatively correlated with LATE‐NC at false discovery rate (FDR) <0.05 (Figure 1A). Downregulated pathways (Figure 1B) included regulation of membrane potential, synaptic transmission, and vesicle‐mediated transport, while upregulated pathways (Figure 1C) involved microtubule‐based movement and cilium assembly. By contrast, despite a much larger sample size, we did not observe any associated genes in the DLPFC (all FDR>0.05)—a region involved only in advanced LATE‐NC. Targeted analysis of the amygdala LATE‐NC‐associated genes in the DLPFC (nominal *p* <0.05, with a consistent direction of association) revealed 8 positively associated and 28 negatively associated genes (Figure 2A‐C). The LATE‐NC‐associated genes downregulated in both amygdala and DLPFC captured synaptic function and vesicle transport pathways, reflecting shared neuronal/synaptic disruption across brain regions in LATE‐NC.

**Conclusions:**

Our findings highlight distinct molecular signatures of LATE‐NC in the amygdala, including upregulation of microtubule‐related pathways and downregulation of neuronal/synaptic genes. Interestingly, this LATE‐NC signature was much weaker in the neocortex, underscoring the critical importance of brain‐region‐specific multi‐omic studies in neurodegeneration. Our results provide one of the first large‐scale molecular atlas of LATE‐NC from the limbic region, providing foundations for disease models, biomarker discovery, and therapeutic approaches.